# Supplementary material for: Epidemiological Study of Autoimmune Bullous Dermatoses in Northeastern Romania
Source: Diagnostics (Basel). 2023 Dec 26;14(1):57. doi: 10.3390/diagnostics14010057 (PMC10871116; doi:10.3390/diagnostics14010057)
Supplement: Supplementary file 1 [file diagnostics-14-00057-s001.zip › diagnostics-2702641-supplementary.pdf]

**Table S1.** Incidence rates of PV in different countries of the world.

| **Country** | **Region** | **Time Period (y)** | **Annual Incidence Rate (/Million)** |
|---|---|---|---|
| Botswana [17] | Gaborone, Kanye, Mochudi, Lobatse, Mahalapye | 2008–2015 | 1.7 (0.9 [PF]; 0.8 [PV] ) |
| Bulgaria [18] | Sofia | 1980–1995 | 4.7 |
| Croatia [19] | Zagreb | 2005–2010 | 3.7 |
| Finland[20] | Nationwide | 1969–1978 | 0.8 |
| France [21] | Midi-Pyrénées region | 2002–2006 | 2.7 |
| Germany [22] | lower Franconia | 2001–2002 | 0.5 (PV) |
| Greece[23] | Thessaloniki | 1985–2004 | 8.0 (PV) |
| India [24] | Thrissur District | 2001 | 4.4 |
| Israel [25] | Jerusalem | 1952–1972 | 16.1 (PV) |

| | | | |
|---|---|---|---|
| Israel [26] | Haifa | 2000–2015 | 7.2 |
| Iran [27] | Tehran | 1984–2003 | 16.0 (Tehran District), 10.0 (Iran) |
| Italy [28] | Sicily | 1982–1996 | 6.0 |
| North Macedonia [29] | Skopje | 1990–2004 | 4.4 |
| Poland [30] | Podlaskie Province | 2001–2015 | 3.7 |
| Romania [14] | Northwestern Romania | 2001–2007 | 4.0 |
| Serbia [31] | Vojvodina | 1990–2002 | 6.6 |
| Spain [32] | Canary Islands | 2004–2017 | 5.9 |
| Switzerland [33] | Nationwide | 2001–2002 | 0.6 |
| Taiwan [34] | Nationwide | 2002–2009 | 4.7 |
| Taiwan [35] | Nationwide | 2010–2015 | 4.0 |

| | | | |
|---|---|---|---|
| Tunisia [36] | Northern Tunisia | 1997–2007 | 8.6 |
| Turkey [37] | All regions of the country | 2013–2014 | 4.7 |
| United Kingdom [38] | Nationwide | 1996–2006 | 6.8 (PV) |
| United States [39] | Connecticut | 1972–1977 | 4.2 (general population), 32 (among Jews) |
